# Radiomics features from perihematomal edema for prediction of prognosis in the patients with basal ganglia hemorrhage

**DOI:** 10.3389/fneur.2022.982928

**Published:** 2022-11-08

**Authors:** Peng Zhou, Quanye Sun, Gesheng Song, Zexiang Liu, Jianfeng Qi, Xuhui Yuan, Xu Wang, Shaofeng Yan, Jianyang Du, Zhengjun Dai, Jianjun Wang, Shaoshan Hu

**Affiliations:** ^1^Department of Neurosurgery, The First Affiliated Hospital of Shandong First Medical University & Shandong Provincial Qianfoshan Hospital, Jinan, China; ^2^Research Center of Translational Medicine, Central Hospital Affiliated to Shandong First Medical University, Jinan, China; ^3^Department of Radiology, The First Affiliated Hospital of Shandong First Medical University & Shandong Provincial Qianfoshan Hospital, Jinan, China; ^4^Department of Neurosurgery, Shandong Provincial Hospital Affiliated to Shandong First Medical University, Jinan, China; ^5^Scientific Research Department, Huiying Medical Technology Co., Ltd, Beijing, China; ^6^Department of Neurosurgery, Emergency Medicine Center, Hangzhou Medical College, Zhejiang Provincial People's Hospital, Hangzhou, China

**Keywords:** radiomics, perihematomal edema, machine learning model, prognosis, basal ganglia hemorrhage

## Abstract

**Objective:**

We developed and validated a clinical-radiomics nomogram to predict the prognosis of basal ganglia hemorrhage patients.

**Methods:**

Retrospective analyses were conducted in 197 patients with basal ganglia hemorrhage (training cohort: *n* = 136, test cohort: *n* = 61) who were admitted to The First Affiliated Hospital of Shandong First Medical University (Shandong Provincial Qianfoshan Hospital) and underwent computed tomography (CT) scan. According to different prognoses, patients with basal ganglia hemorrhage were divided into two groups. Independent clinical risk factors were derived with univariate and multivariate regression analysis. Radiomics signatures were obtained using least absolute shrinkage and selection operator. A radiomics score (Rad-score) was generated by 12 radiomics signatures of perihematomal edema (PHE) from CT images that were correlated with the prognosis of basal ganglia hemorrhage patients. A clinical-radiomics nomogram was conducted by combing the Rad-score and clinical risk factors using logistic regression analysis. The prediction performance of the nomogram was tested in the training cohort and verified in the test cohort.

**Results:**

The clinical model conducted by four clinical risk factors and 12 radiomcis features were used to establish the Rad-score. The clinical-radiomics nomogram outperformed the clinical model in the training cohort [area under the curve (AUC), 0.92 vs. 0.85] and the test cohort (AUC, 0.91 vs 0.85). The clinical-radiomics nomogram showed good calibration and clinical benefit in both the training and test cohorts.

**Conclusion:**

Radiomics features of PHE in patients with basal ganglia hemorrhage could contribute to the outcome prediction. The clinical-radiomics nomogram may help first-line clinicians to make individual clinical treatment decisions for patients with basal ganglia hemorrhage.

## Introduction

Spontaneous intracerebral hemorrhage (ICH) accounts for 10% of all strokes and has a high mortality rate of ~40% (1). The basal ganglia are the most common site of ICH. Globally, ICH leads to 2.8 million deaths per year (2, 3), and only 12–39% of ICH patients could live independently without disabilities (4). Because ICH usually leads to death, morbidity, and disability, early and accurate prediction of clinical prognosis is important to guide the development of clinical treatment plans and observe the effect of treatment.

Perihematomal edema (PHE) is caused by damage to the blood-brain barrier (BBB) and neuronal ion channel disruption and is an important secondary injury following ICH (5, 6). It is the primary cause of increased intracranial pressure, brain hernia, and death in ICH patients and contributes to poor clinical prognosis (7, 8). Many studies have shown that increased PHE volume around the hematoma after ICH was an independent risk factor in ICH patients for poor prognosis (9–12). Computed tomography (CT) is the most common examination method for diagnosing PHE. During the early stage, the characteristics of PHE are not typical in CT images, and accurate interpretations rely on radiologists' experience (13–15). It is difficult for clinicians to quantify early cerebral edema following ICH. Therefore, developing a more objective and convenient method for volume and severity assessment of the PHE in CT scans will significantly benefit prognosis predictions and could contribute to clinical intervention decision-making.

Radiomics is a rapidly developing method based on computer-aided detection or diagnosis and combines quantitative image analysis and machine learning algorithms (16–19). Radiomics overcomes the limitation of image interpretation, which usually relies on the experience of doctors (20, 21). At present, radiomics is used primarily for screening and quantitative analysis of the most valuable imaging features, which are used to develop machine learning models for either diagnosis or prognosis prediction (22–24). Although previous studies have suggested that PHE is a predictor of functional outcomes in ICH patients, the impact of PHE on the prognosis of ICH is controversial (25–28). Currently, the association between the radiomics features of PHE in CT scans and the outcome of ICH patients remains unclear.

In this study, we aimed to establish and validate a combined nomogram for predicting the prognosis of basal ganglia hemorrhage patients using the radiomics features of PHE and clinical characteristics.

## Materials and methods

### Collection and selection of patient data

**Figure 1:**
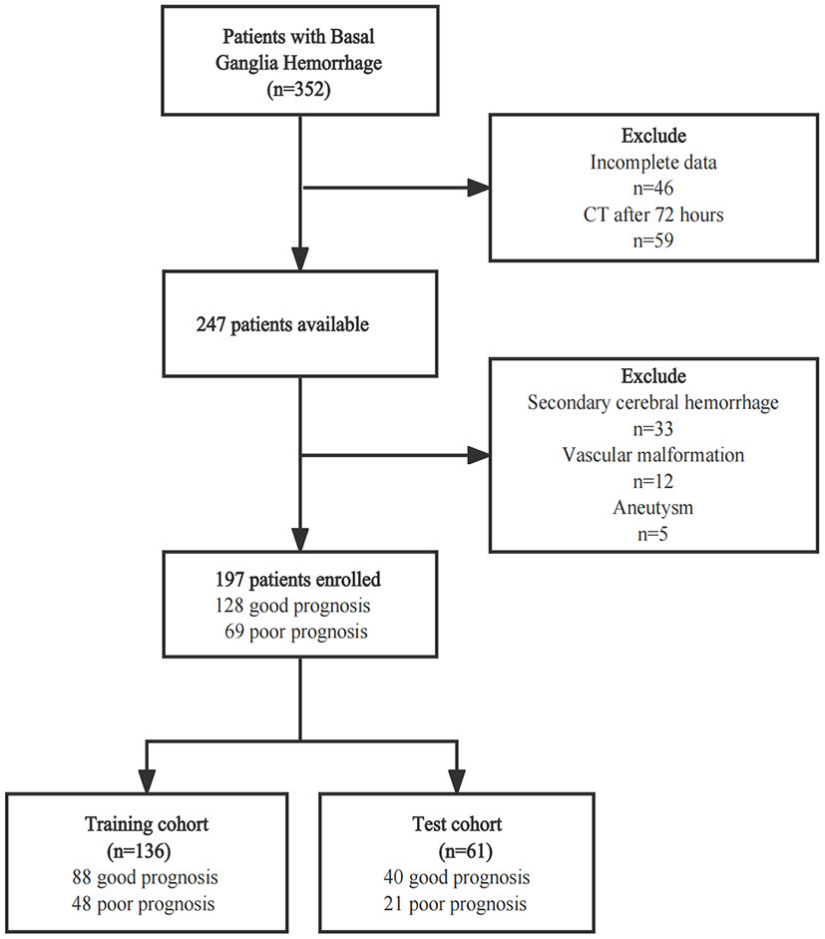
The flow chart of patients' selection.

This single-center retrospective study was approved by the Medical Ethics Committee of The First Affiliated Hospital of Shandong First Medical University (Shandong Provincial Qianfoshan Hospital), and the informed consent was waived. 352 ICH patients were recruited between January 2016 and March 2022. For this study, the inclusion criteria were as follows: (1) patients aged over 18 years with a spontaneous ICH in the basal ganglia; (2) CT examination performed within 72 h of disease onset; (3) admission assessment using the National Institute of Health Stroke Scale (NIHSS). Patients with the following conditions were excluded: (1) patients with a tumor, vascular malformation, aneurysm, or trauma, and those who had undergone thrombolytic therapy or cerebral arteriovenous thrombosis; (2) patients pretreated with anticoagulants or those with coagulopathy; (3) pregnant women; (4) patients with multiple organ failure. Finally, a total of 197 patients were enrolled. This patient database was divided into a training cohort (*n* = 136) and a test cohort (*n* = 61) at a 7:3 ratio, with the random seed of 186 (Figure 1). The Glasgow Outcome Scale (GOS) was used for assessing the clinical outcome of patients when they were discharged from the hospital, which was usually 7–10 days after admission. A GOS score of 4–5 represented a good prognosis, while a score of 1–3 was regarded as a poor prognosis (Figure 1).

### Images acquisition and region of interest segmentation

The first CT scans of patients after ICH onset were acquired on two types of CT scanners (Discovery CT750 HD and Optima CT660, General Electric Company, USA) using standardized scanning protocols: tube voltage and current of 120 kV and 350 mA, the field of view of 32 cm, matrix size of 512 × 512, and slice thickness of 5 mm. The scanning range was from the skull base to the cranium.

The region of interest (ROI) segmentation of the PHE and hematoma were performed by a neuroradiologist with 10 years of experience using Radcloud (Huiying Medical Technology Co., Ltd., China). The validation of segmentation results was conducted by a senior neuroradiologist with 20 years of experience in 19 randomly selected patients. All radiologists were blind to the clinical information of the patients.

### Feature extraction and selection

**Figure 2:**
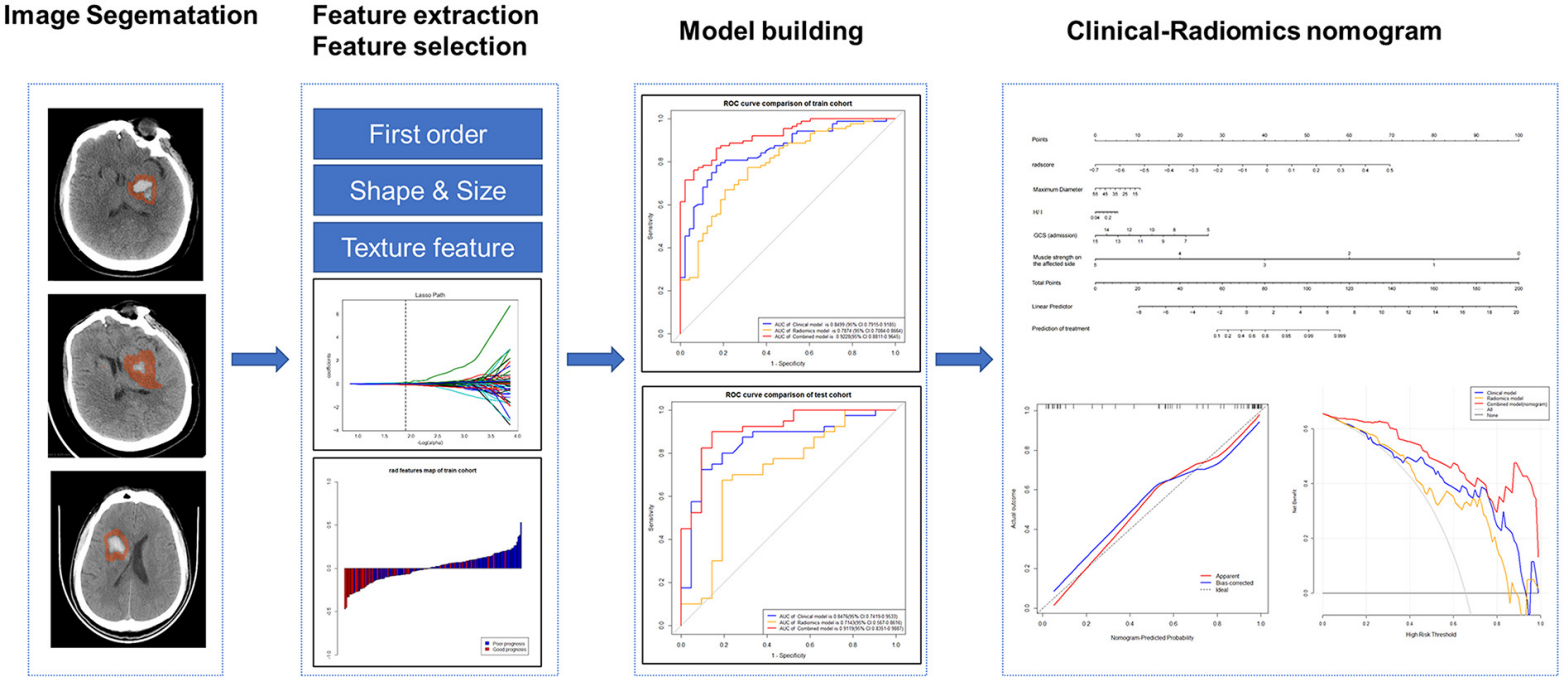
The workflow of the radiomics analysis.

Using the Radcloud platform, we extracted 1,409 quantitative imaging features of PHE from the CT images. These features contained first-order statistics and texture, shape, and size features. The feature extraction was conducted using a “pyradiomics” package (https://pyradiomics.readthedocs.io/en/latest/). We first conducted the intraclass correlation coefficient test in 19 patients, and 1,225 features with a *p* > 0.75 were screened for the subsequent analysis. The variance threshold method reduced the number of features to 1,178, of which 131 were retained after applying the SelectKBest method. Finally, using the least absolute shrinkage and selection operator (LASSO) regression model, 12 radiomics signatures were selected for machine model building (Figure 2). The features of hematoma were extracted in the same way as for PHE and were combined with the features of PHE for selection. The combined feature selection was conducted using the variance threshold (variance threshold = 0.8), SelectKBest (*p* < 0.05), and the LASSO, and 12 combined features were obtained (Supplementary materials). PHE volume and hematoma volume were calculated using the Radcloud platform.

### Machine learning model building

Clinical characteristics were screened using univariate and multivariate logistic regression analyses. Factors with a *p* < 0.05 were considered single risk factors for prognosis in basal ganglia hemorrhage patients by univariate logistic regression analyses in the training cohort. These single factors were then analyzed by multivariate logistic regression analyses, and factors with a *p* < 0.1 were considered independent risk factors for prognosis. Using these factors, we built a clinical model using logistic regression in the training cohort and verified in the test cohort.

PHE volume-clinical model was conducted with the PHE volume and the four independent clinical features by logistic regression in the training cohort and verified in the test cohort.

The radiomics score (Rad-score) was calculated for each patient by a formula using the selected 12 radiomics signatures. The rad-score formula was obtained as follows: Rad-score = $\alpha +\sum_{1}^{\text{i}}\beta iXi$, and α is the intercept (α = 0.647), βi is the value of radiomics feature; Xi is the corresponding coefficient (Supplementary materials).

Based on the selected features of PHE, we constructed radiomics models with three classifiers, including Logistic Regression (LR), Decision tree (DT), and Support Vector Machine (SVM). The effectiveness of the model was improved using the validation method.

The clinical-radiomics nomogram was derived using the rad-score and the independent clinical risk factors in the training cohort and verified in the test cohort using logistic regression analysis.

The PHE-hematoma-clinical model was conducted with four independent clinical risk factors and the 12 selected combined radiomics features using LR analysis in the training cohort and verified in the test cohort (Supplementary materials).

### Model evaluation

The receiver operating characteristic (ROC) curve was used to assess the predictive efficacy of machine learning models in the training and test cohort. Delong test was used to evaluate the differences of the area under curves (AUCs). The nomogram was evaluated by calibration curves and Hosmer-Lemeshow test in the training and test cohorts. Decision curve analysis (DCA) was conducted to determine the clinical benefit of the machine learning models by calculating the net benefits at different threshold probabilities.

### Statistical analysis

Statistical analyses were conducted with R software (version 3.4.4) and the SPSS software (version 22.0). Clinical characteristics are described as medians (interquartile ranges) or means ± standard deviations according to the results of the Shapiro-Wilk test. Categorical data, such as sex, are expressed as percentages. The chi-squared test, Fisher's exact test, and Mann-Whitney U test were used for univariate analysis. A *p* < 0.05 was considered statistically significant.

## Results

### Characteristics of basal ganglia hemorrhage patients

There were no significant differences in clinical characteristics between the training and test cohorts (Table 1). Univariate analysis indicated that maximum diameter, hematoma/intracranial diameter (H/I), Glasgow Coma Scale (GCS) score, Mg (magnesium), hematoma volume, D-dimer (DD2), NIHSS score, and muscle strength on the affected side were potential risk factors for prognosis in basal ganglia hemorrhage (Table 2, *p* < 0.05). These eight clinical features were subsequently analyzed using multivariate logistic regression and obtained four independent predictors of prognosis: GCS score (*p* = 0.013), muscle strength on the affected side (*p* < 0.001), hematoma volume (*p* = 0.092), and DD2 (*p* = 0.047).

**Table 1:** Patients' characteristics in the training and test cohorts.

**Variables**	**Training cohort (*n* = 136)**	**Test cohort (*n* = 61)**	***P***
Age, years	60 ± 11.8	59.5 ± 12.9	0.372
Sex (male), *n* (%)	78 (57)	44 (72)	0.057
Muscle strength on the affected side	3 (1,4)	2 (1,4)	0.484
Minimum diameter, mm	16.81 (14.51, 20.98)	18.52 (13.05, 21.65)	0.088
Maximum diameter, mm	31.36 ± 10.77	29.93 ± 8.5	0.718
Roundness, mm	12.2 (6.45, 19.5)	11.4 (5.7, 18.9)	0.337
Intracranial diameter, mm	127.31 ± 7.28	128.6 ± 7.5	0.485
Hematoma diameter, mm	19.94 (15.65, 24.29)	21.65 (14.67, 26.76)	0.275
H/I	0.16 ± 0.05	0.17 ± 0.05	0.189
GCS score	11(9,13)	10 (9,13)	0.879
Alkaline phosphatase	75 (64, 90.15)	71.6 (62.6, 88)	0.751
K, mmol/L	3.88 (3.52, 4.07)	3.91 (3.57, 4.3)	0.413
Na, mmol/L	141.06 (137, 143)	141.1 (138, 143)	0.307
Ca, mmol/L	2.23 (2.11, 2.33)	2.21 (2.16, 2.29)	0.799
Mg, mmol/L	0.88 (0.84, 0.91)	0.89 (0.85, 0.92)	0.095
Blood glucose at admission, mmol/L	5.97 (4.94, 6.91)	5.9 (5.06, 7.3)	0.710
WBC, 10^9^/L	7.66 (6.38, 9.49)	8.09 (6.06, 9.58)	0.884
Neutrophils,10^9^/L	6.03 (4.44, 7.68)	5.77 (4.38, 7.96)	0.613
Lymphocyte,10^9^/L	1.16 (0.77, 1.55)	1.3 (0.78, 1.73)	0.448
NLR	5.08 (3.14, 9.63)	4.54 (2.77, 7.36)	0.471
Hb, g/L	137.2 ± 15.46	137.98 ± 14.57	0.842
HCT	0.41 ± 0.04	0.42 ± 0.04	0.497
RDW–CV	12.5 (12,13)	12.7 (12.1, 13.2)	0.346
PLT, 10^9^/L	218 (185, 251.75)	213 (171, 265)	0.299
PDW, fL	12 (10.47, 13.43)	11.9 (10.7, 12.9)	0.569
PT, sec	11.2 (10.7, 11.6)	11.3 (10.6, 11.7)	0.914
INR	0.96 (0.91, 1.01)	0.97 (0.90, 1.03)	0.689
APTT, sec	25.4 (23.45, 27.13)	25.6 (21.73, 29.43)	0.456
TT, sec	17.05 (16.38, 17.8)	17.1 (16.5, 17.9)	0.621
DD2, mg/L	0.35 (0.2, 0.69)	0.35 (0.22, 0.75)	0.657
Systolic pressure at hospital admission	164 ± 27.4	162.3 ± 23.6	0.745
Diastolic pressure at hospital admission	95.4 ± 15.8	93 ± 13.0	0.054
Hematoma volume, mL	16.64 (9.9, 27.83)	15.23 (6.47, 23.38)	0.149
NIHSS score	7 (4,11)	6 (4,11)	0.429
First CT (hour)	19 (12,30)	20 (12,33)	0.672

H/I, hematoma/intracranial diameter; GCS, Glasgow Coma Scale; NIHSS, National Institute of Health stroke scale. Data are shown as median (interquartile ranges) or mean ± standard deviations.

**Table 2:** Univariate analyses of predictors of prognosis in training cohorts.

**Variables**	**Good prognosis** **(*n* = 88)**	**Poor prognosis** **(*n* = 48)**	***P***
Age, years	62.5 ± 13.1	59.1 ± 10.1	0.203
Sex (male), *n* (%)	52 (59)	26 (54)	0.709
Muscle strength on the affected side	4 (2-4)	0 (0–2)	< 0.001
Minimum diameter, mm	16.63 (13.84, 18.79)	17.66 (15.13, 22.37)	0.060
Maximum diameter, mm	29.81 ± 9.93	34.22 ± 11.74	0.021
Roundness, mm	10.3 (6.31, 17.44)	13.48 (6.9, 21.93)	0.116
Intracranial diameter, mm	127.74 ± 7.71	126.53 ± 6.41	0.355
Hematoma diameter, mm	8.16 (14.9, 23.37)	21.04 (17.14, 25.64)	0.067
H/I, median	0.15 ± 0.06	0.17 ± 0.05	0.048
GCS score	12 (10,13)	9 (7.75, 10)	<0.001
Alkaline phosphatase	75.55 (64.38, 90.15)	72.3 (63.25, 88.75)	0.609
K, mmol/L	3.84 (3.54, 4.05)	3.9 (3.49, 4.11)	0.460
Na, mmol/L	141.18 ± 3.95	140.86 ± 3.95	0.651
Ca, mmol/L	2.22 ± 0.1	2.25 ± 0.11	0.104
Mg, mmol/L	0.91 ± 0.1	0.86 ± 0.1	0.009
Blood glucose at admission, mmol/L	6.26 (5.46–7.15)	5.36 (4.79–6.02)	0.163
WBC, 10^9^/L	7.5 (6.32, 8.95)	8.16 (6.49, 9.72)	0.084
Neutrophils,10^9^/L	5.74 (4.42, 7.6)	6.22 (4.62, 8.29)	0.084
Lymphocyte,10^9^/L	1.17 (0.8, 1.54)	1.09 (0.75, 1.61)	0.432
NLR	5 (3.04, 8.47)	5.6 (3.49, 10.89)	0.344
Hb, g/L	138.35 ± 14.76	135.08 ± 16.62	0.240
HCT	0.41 ± 0.04	0.4 ± 0.05	0.108
RDW–CV	12.9 (12.0–13.4)	12.2 (11.9–13.0)	0.331
PLT, 10^9^/L	219.5 (188.5, 256.5)	215.5 (167.25, 248)	0.595
PDW, fL	11.75 (10.47, 13.53)	12.2 (10.45, 13.25)	0.917
PT, sec	11.1 (10.7, 11.43)	11.2 (10.67, 11.7)	0.080
INR	0.96 (0.92, 1)	0.96 (0.9, 1.02)	0.149
APTT, sec	25.4 (23.7, 26.85)	25.25 (23.03, 27.45)	0.069
TT, sec	17.2 (16.6, 17.83)	16.8 (16.17, 17.4)	0.872
DD2, mg/L	0.29 (0.18, 0.54)	0.5 (0.24, 0.88)	0.027
Systolic pressure at hospital admission	165.6 ± 27.5	161.3 ± 27.1	0.727
Diastolic pressure at hospital admission	96.3 ± 16.4	93.8 ± 14.8	0.934
Hematoma volume, mL	13.97 (8.18, 24.98)	26.82 (16.23, 35.1)	<0.001
NIHSS score	5 (3,8)	10 (7,14)	<0.001
First CT (hour)	20 (12, 29.25)	19 (12, 31.25)	0.655

H/I, hematoma/intracranial diameter; GCS, Glasgow Coma Scale; NIHSS, National Institute of Health stroke scale. Data are shown as median (interquartile ranges) or mean ± standard deviations.

### Clinical and radiomics models for prognosis prediction in patients with basal ganglia hemorrhage

**Figure 3:**
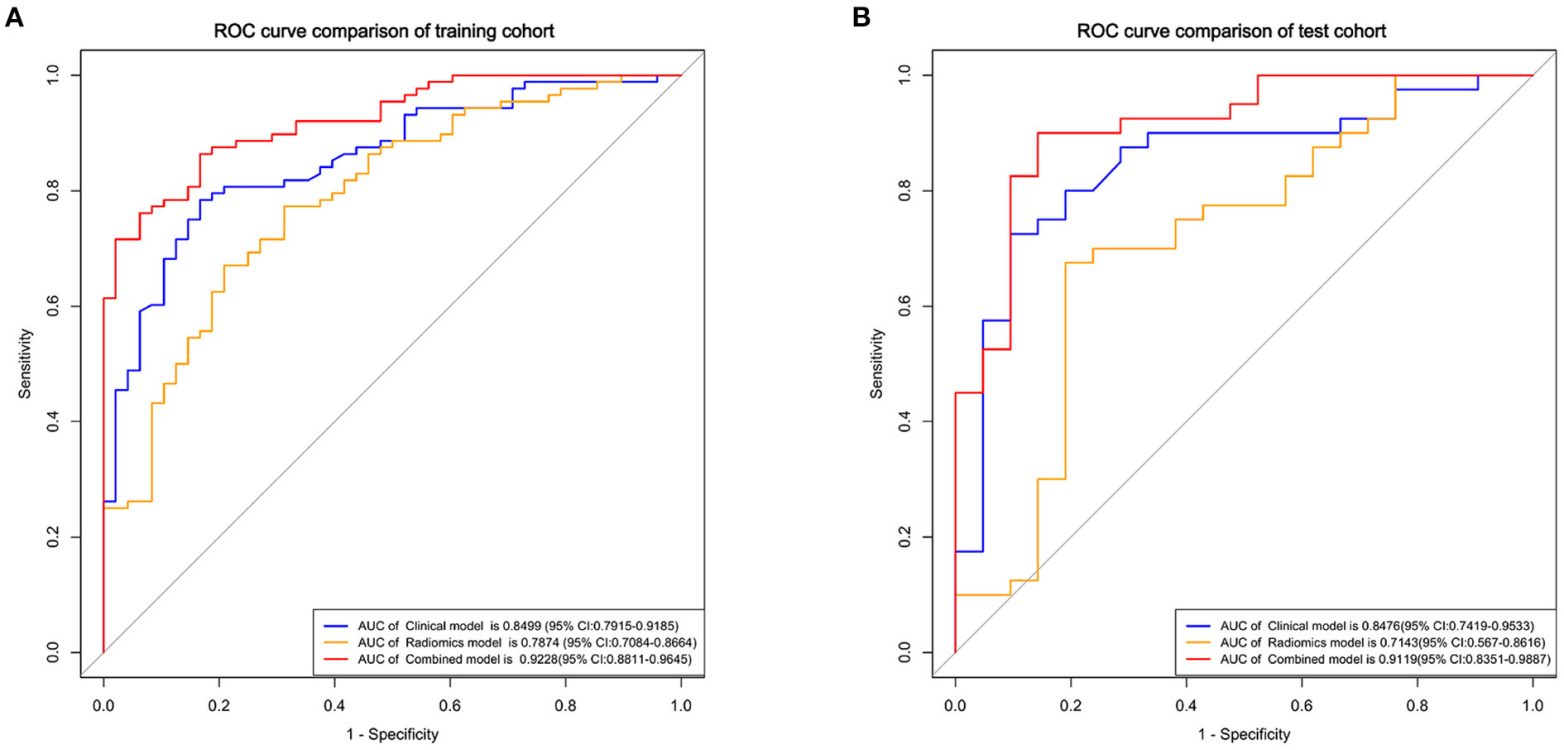
Receiver operating characteristic (ROC) curves of the clinical, radiomics, and combined models for predicting the prognosis of basal ganglia hemorrhage patients in the training **(A)** and test cohorts **(B)**.

The clinical model comprising four independent risk factors had an AUC of 0.85 [95% confidence interval (CI), 0.79–0.92] in the training cohort, and the AUC was 0.85 (95% CI, 0.74–0.95) in the test cohort (Figure 3).

The radiomics model comprised three different classifiers using the 12 radiomics features. As shown in Supplementary Figure 2, in the training cohort, the AUC of the SVM model was 0.79 (95% CI, 0.71–0.87), the AUC of the LR model was 0.79 (95% CI, 0.71–0.87), and the AUC of the DT model was 0.74 (95% CI, 0.67–0.81). In the test cohort, the AUC of the SVM model was 0.70 (95% CI, 0.56–0.85), the AUC of the LR model was 0.71 (95% CI, 0.57–0.86), and the AUC of the DT model was 0.67 (95% CI, 0.50–0.76). The LR model got better performance than the SVM and DT models (Supplementary Table 1). The results of Delong test showed that although the clinical model had higher AUCs than the LR radiomics model, no significant difference was found between these models in the training cohort (*p* = 0.152) and the test cohort (*p* = 0.159).

To verify whether the PHE volume contributes to the enhanced prediction of prognosis, we combined the four independent clinical risk factors and the PHE volume to build a model to predict the prognosis of basal ganglia hemorrhage patients. Results showed that PHE volume-clinical model had an AUC of 0.91 (95% CI, 0.87–0.96) in the training cohort and an AUC of 0.84 (95% CI, 0.74–0.95) in the test cohort (Supplementary Figure 3). This PHE volume-clinical model did not show a better performance than the clinical model in prognosis prediction.

### Development of the clinical-radiomics nomogram

Using the four clinical independent risk factors and the Rad-score, a clinical-radiomics combined model was built using a logistic regression classifier. As shown in Figure 3, the AUC of the combined model was 0.92 (95% CI, 0.88–0.96) in the training cohort and 0.91 (95% CI, 0.84–0.99) in the test cohort. The clinical-radiomics model showed a better performance in prognosis prediction than the clinical (*p* = 0.006, Delong test) and radiomics models (*p* < 0.001, Delong test) in the training cohort. In the test cohort, although the clinical-radiomics model did not perform significantly differently from the clinical (*p* = 0.203), but had a better performance in prognosis prediction than the radiomics model (*p* = 0.002).

**Figure 4:**
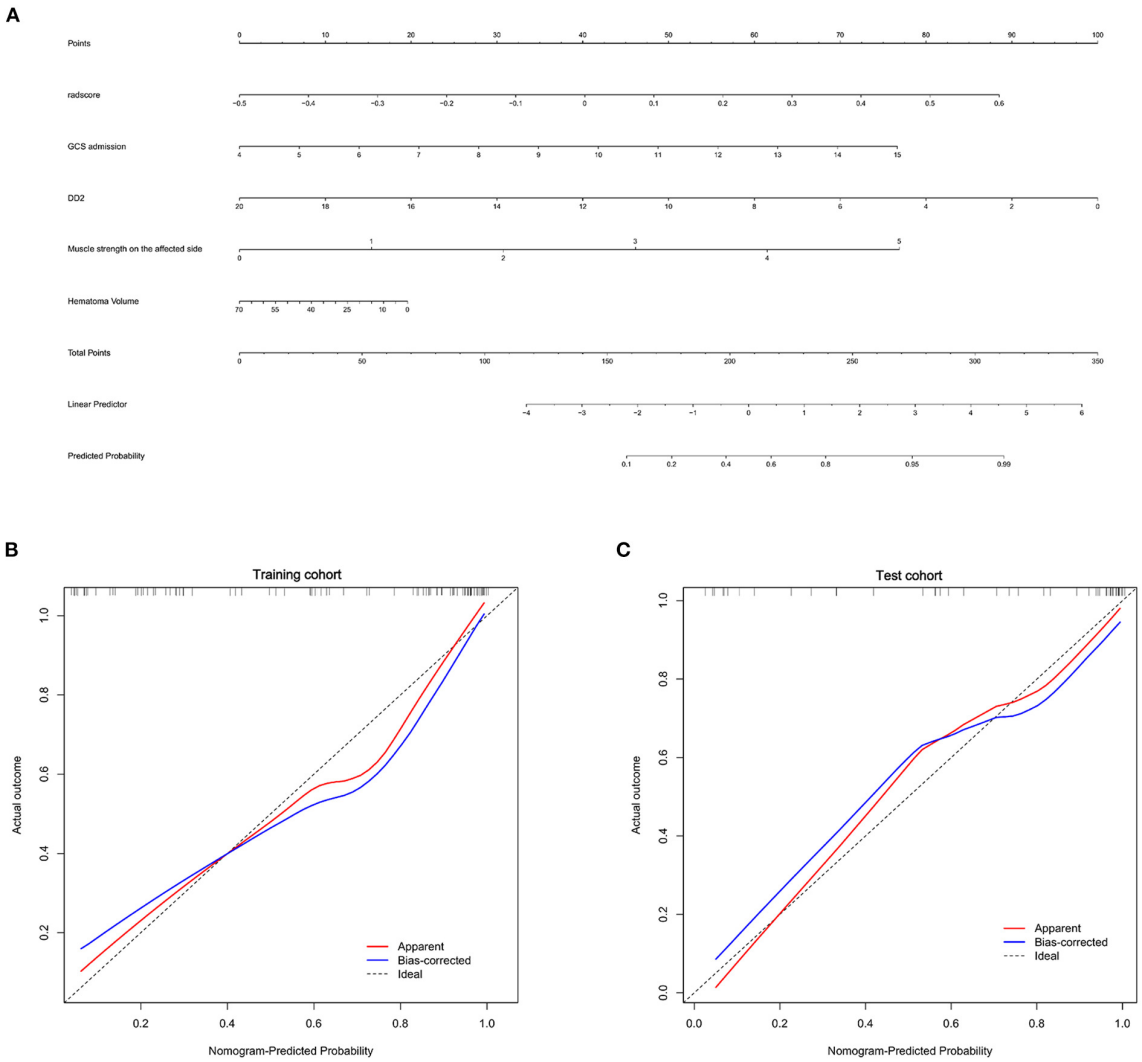
Nomogram for prognosis prediction and the calibration of the nomogram in the patients with basal ganglia hemorrhage. **(A)** The clinical-radiomics nomogram for the prediction of prognosis of basal ganglia hemorrhage patients. Calibration curves of the radiomics nomogram in the training **(B)** and test cohorts **(C)**.

The clinical-radiomics nomogram for determining the prognosis of basal ganglia hemorrhage patients was shown in Figure 4. The calibration curves indicated the prediction probabilities of the nomogram were well-aligned with the actual outcome in both the training (*p* = 0.154) and test cohorts (*p* = 0.860).

We also developed a PHE-hematoma-clinical model using 12 combined radiomics features of PHE and hematoma and the four independent clinical features for prognosis prediction. The PHE-hematoma-clinical model showed AUCs of 0.91 and 0.90 in the training and test cohort (Supplementary Figure 4). This PHE-hematoma-clinical model did not show a better performance in prognosis prediction than the clinical-radiomics model (Supplementary Table 2).

**Figure 5:**
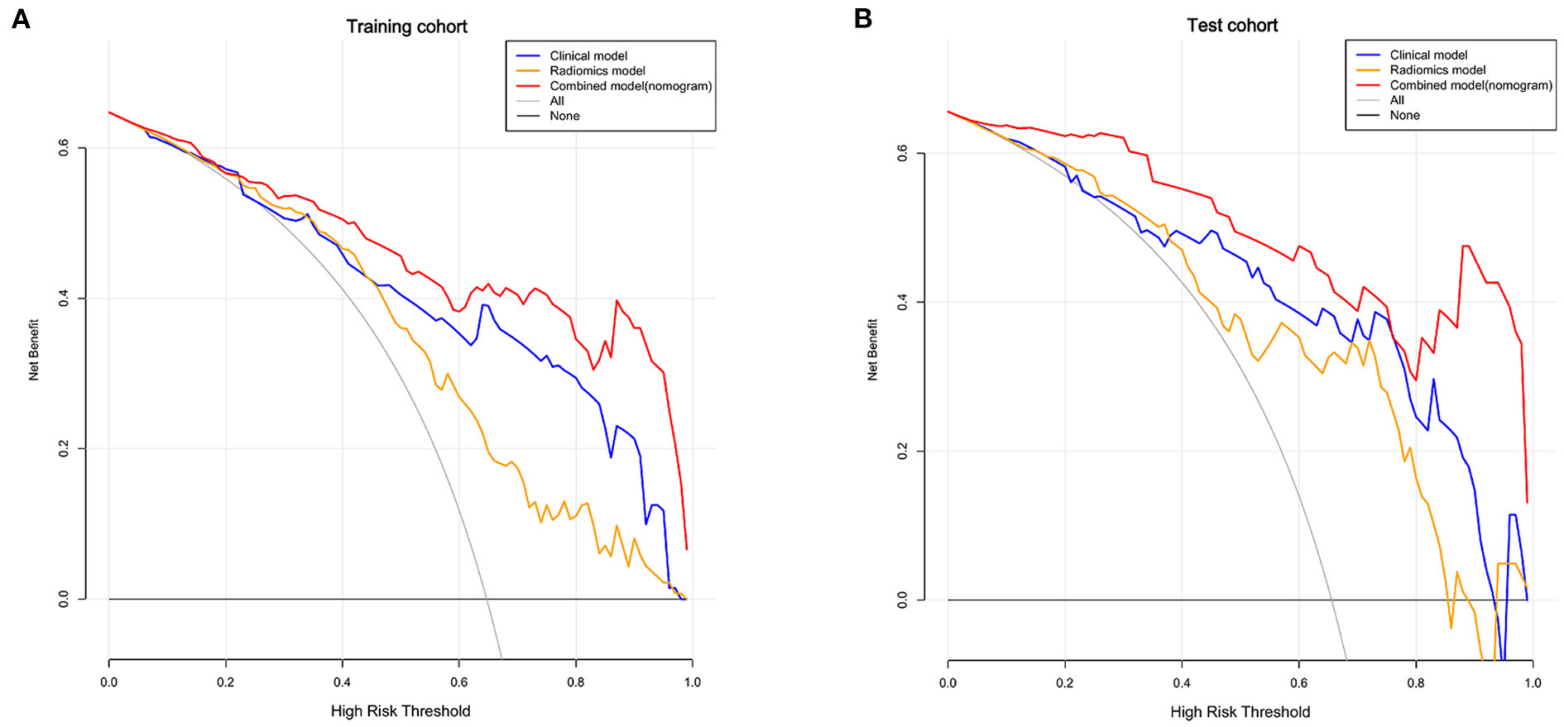
Decision curves of the clinical, radiomics, and combined models in the training **(A)** and test cohorts **(B)**. The X-axis indicates the threshold probability and Y-axis indicates the net benefit. The gray line indicates the net benefit of all patients would have a good prognosis; the black line indicates the net benefit of no patients would have a good prognosis, namely, all patients would have a poor prognosis. The red, blue and yellow lines represent the net benefit of the clinical-radiomics nomogram, clinical model, and radiomics model, respectively.

Finally, we used DCA analysis to compare the clinical benefits of different prediction models. As shown in Figure 5, the decision curves graphically displayed that the clinical-radiomics model had a better benefit than the clinical and radiomics models, indicating the superiority of the combined model.

## Discussion

In this research, we confirmed that radiomics features of PHE from CT images combined with clinical features are valuable for prognosis prediction of patients with basal ganglia hemorrhage. Compared with the clinical model, the clinical-radiomics model showed a better performance for prognosis prediction. The nomogram derived from this clinical-radiomics model will enable first-line clinicians to evaluate ICH patients and develop individual treatment strategies without relying on substantial experience in diagnostic imaging.

At present, most clinicians predict the prognosis of ICH patients using clinical characteristics. Therefore, we first analyzed the clinical data of patients. Statistical results showed that DD2, GCS score, hematoma volume, and muscle strength on the affected side were independent predictors of prognosis in basal ganglia hemorrhage patients. The GCS has been widely used in clinical research to assess and calculate the level of consciousness of patients (29–31). In line with our results, the GCS score has been shown to be strongly associated with the outcome of basal ganglia hemorrhage patients and is an independent predictor of critical care (32, 33). Similar to the previous studies, plasma DD2 could predict poor outcome and mortality in ICH patients (34–36). We used these four risk factors to build a clinical model using logistic regression and yielded in the test cohort (AUC: 0.85), which indicated that the clinical model does not provide sufficient accuracy for predicting prognosis in basal ganglia hemorrhage patients. Combining these clinical features with other variables would likely improve the predictive ability of this machine learning model.

PHE is associated with secondary injury in ICH (5, 36). Volbers et al. (37) showed that the volume of PHE is an independent predictive factor for ICH patients at 90 days post-onset. However, it has controversy for the connection between PHE and the ICH patients' outcome (38). Loan et al. (39) reported the volume of PHE was not independently associated with death or dependence 1 year after ICH, as well as the total volume of ICH and PHE are independent risk factors. As shown in this research, the AUC of PHE volume-clinical model was 0.84 in the test cohort, suggesting that PHE volume could not improve the prediction ability of the clinical model. Radiomics features of PHE may have a better-discriminating efficacy for the prognosis of basal ganglia hemorrhage patients.

Although radiomics features enable the quantification of medical imaging characteristics, they are difficult to reproduce and validate according to published studies because of the lack of standardized definitions. The Image Biomarker Standardization Initiative formed in 2016 allows the validation of different radiomics software (40). This finding contributed to the repeatability of medical imaging research. We standardized image processing and feature extraction according to this standard. The Rad-score in our study was derived from 12 radiomics features of PHE in CT images associated with prognoses and included eight texture features (three GLRLM and five GLSZM), three shape features, and one first-order feature.

In our study, the clinical model showed an AUC of 0.85 and radiomics model showed an AUC of 0.71, which indicated the use of only one of these modalities would not offer sufficient accuracy. Thus, we combined these two models, which yielded a much higher AUC (0.91) in the test cohort, which suggested radiomics could improve prognosis prediction in basal ganglia hemorrhage patients.

Since hematoma is a critical factor related to the prognosis of ICH, we added the radiomics features of hematoma for analysis. Results showed an AUC of 0.90 of the PHE-hematoma-clinical model in the test cohort, which did not have a better performance in prognosis prediction compared with the clinical-radiomics model. These results suggested that the clinical-radiomics model contained features of PHE combined with independent clinical features (including hematoma volume) already had a good performance in prognosis prediction of basal ganglia hemorrhage patients. There was no need to add the radiomics features of hematoma for model building.

There are some limitations in our research. First, the data of the patients were obtained from a single center with relatively small sample size. We plan to conduct a multi-center study with a larger patient sample size in the future. Second, we used GOS scores to evaluate the prognosis of basal ganglia hemorrhage patients when they were discharged from the hospital (~7–10 days following ICH onset). We plan to include additional time points, including long-term prognoses, in future studies. Third, the results of DeLong test suggested that the clinical-radiomics model had a higher AUC than the clinical and radiomics models in the test cohort; however, the difference was not significant. We considered the relatively small sample size and segmentation errors might be responsible for this result. Finally, this was a retrospective study, and diagnostic, detection, and evaluation criteria were not standardized. Therefore, several variables could not be analyzed, which may have impacted the predictive ability of the radiomics model.

## Conclusion

We built a clinical-radiomics nomogram (model) comprising clinical independent risk factors and radiomics features of PHE derived from CT images, and this nomogram showed good accuracy for prognosis prediction in basal ganglia hemorrhage patients. Our findings suggested the radiomics features of PHE could contribute to the outcomes prediction of patients with basal ganglia hemorrhage. The clinical-radiomics nomogram may help first-line clinicians in clinical treatment decision-making for basal ganglia hemorrhage patients.

## Data availability statement

The original contributions presented in the study are included in the article/Supplementary material, further inquiries can be directed to the corresponding authors.

## Author contributions

PZ, QS, and GS: writing original draft. ZL, JQ, and JD: organization. ZL, PZ, QS, GS, JQ, and JD: statistical analysis. XY, XW, and SY: data collection. JW, SH, and JD: conceptualization, funding acquisition, and review. JW, SH, PZ, QS, GS, and JD: study design. All authors contributed to the article and approved the submitted version.

## Funding

This study was funded by Clinical Scientific Research Fund of Shandong Medical Association (No. YXH2022ZX02179) and Natural Science Foundation of Shandong Province (No. ZR2020MH153).

## Conflict of interest

Author ZD was employed by Huiying Medical Technology Co., Ltd. The remaining authors declare that the research was conducted in the absence of any commercial or financial relationships that could be construed as a potential conflict of interest.

## Publisher's note

All claims expressed in this article are solely those of the authors and do not necessarily represent those of their affiliated organizations, or those of the publisher, the editors and the reviewers. Any product that may be evaluated in this article, or claim that may be made by its manufacturer, is not guaranteed or endorsed by the publisher.
