# Supplementary material for: Radiomics features from perihematomal edema for prediction of prognosis in the patients with basal ganglia hemorrhage
Source: Front Neurol. 2022 Nov 8;13:982928. doi: 10.3389/fneur.2022.982928 (PMC9680901; doi:10.3389/fneur.2022.982928)
Supplement: Supplementary file 1 [file Table_1.DOCX]

**Supplementary Figure legends**

**Supplementary Figure 1.** (A, B) Determination of the number of factors by the LASSO analysis. C. Feature map in training cohort. D. Feature map in training cohort.

**Supplementary Figure 2.** Receiver operating characteristic (ROC) curves of the Support Vector Machine (SVM), Logistic Regression (LR) and Decision tree (DT) in training cohort (A) and test cohort (B).

**Supplementary Figure 3.** Receiver operating characteristic (ROC) curves of the PHE-volume-clinical model in training cohort (A) and test cohort (B).

**Supplementary Figure 4.** Receiver operating characteristic (ROC) curves of the PHE-hematoma-clinical model in training cohort (A) and test cohort (B).
