# Supplementary material for: Radiomics features from perihematomal edema for prediction of prognosis in the patients with basal ganglia hemorrhage
Source: Front Neurol. 2022 Nov 8;13:982928. doi: 10.3389/fneur.2022.982928 (PMC9680901; doi:10.3389/fneur.2022.982928)
Supplement: Supplementary file 3 [file Table_3.DOCX]

**Supplementary Materials**

**Materials and methods**

#### Image segmentation

The CT images of basal ganglia hemorrhage patients were acquired according to standardized scanning protocols. Since the characteristics of PHE are not typical in CT images during the early stage, segmentation of ROI was done manually by a radiologist with 10 years of experience. who was blinded to the clinical information of the patients. Then all contours were reviewed by a senior radiologist. We then randomly selected 19 patients for intra-observer reproducibility analysis. After extracting 1409 radiomics features from the ROI by Radcloud platform, the interclass correlation coefficient (ICC) was calculated to assess the reproducibility of radiomics features. The results showed that 1225 radiomics features showed ICC > 0.75 in the intra-observer reproducibility analysis. These 1225 radiomics features were screened for the subsequent analysis.

**Feature extraction**

A total of 1409 quantitative imaging features were extracted from CT images with Radcloud platform. According to the image biomarker standardization initiative (IBSI) standard, these features could be grouped into three groups. Group 1 (first order statistics) consisted of 126 descriptors that quantitatively delineate the distribution of voxel intensities within the CT image through commonly used and basic metrics. Group 2 (shape- and size-based features) contained 14 three-dimensional features that reflect the shape and size of the region. Group 3 (texture features) contained 525 textural features calculated from three types including Gray Level Cooccurence Matrix (GLCM), Gray Level Run Length Matrix (GLRLM) and Gray Level Size Zone Matrix (GLSZM).

**Rad-score formula of PHE**

The rad-score formula was obtained as follows: Rad-score=0.091386611*(wavelet-LLH_glszm_SmallAreaHighGrayLevelEmphasis)-0.078689798*(original_shape_LeastAxisLength)-0.054904602*(exponential_firstorder_TotalEnergy)-0.030206978*(original_shape_Maximum2DDiameterSlice)-0.101915608*(wavelet-LLH_glszm_LargeAreaLowGrayLevelEmphasis)-0.007137700*(original_glszm_LargeAreaHighGrayLevelEmphasis)-0.015295464*(wavelet-HHL_glszm_SizeZoneNonUniformity)+0.115923227*(exponential_glrlm_GrayLevelNonUniformity)+4.24E-17*(gradient_glrlm_GrayLevelNonUniformity)+0.024394049*(original_glrlm_GrayLevelNonUniformity)-0.062859601*(wavelet-HLH_glszm_GrayLevelNonUniformity)+0.018957420*(original_shape_Maximum2DDiameterRow).

**Supplementary Table 1** Performance of three radiomics models

|  |  | AUC | 95%CI | Accuracy | Sensitivity | Specificity |
| --- | --- | --- | --- | --- | --- | --- |
| Training cohort | LR | 0.79 | 0.71–0.87 | 0.74 | 0.82 | 0.61 |
|  | SVM | 0.79 | 0.71–0.87 | 0.74 | 0.74 | 0.74 |
|  | DT | 0.74 | 0.67–0.81 | 0.77 | 0.74 | 1.00 |
| Test cohort | LR | 0.71 | 0.57–0.86 | 0.69 | 0.83 | 0.46 |
|  | SVM | 0.70 | 0.56–0.85 | 0.61 | 0.81 | 0.43 |
|  | DT | 0.67 | 0.52–0.82 | 0.67 | 0.68 | 0.57 |

**Supplementary Table 2**  Performance of different models

|  |  | AUC | 95%CI | Accuracy | Sensitivity | Specificity |
| --- | --- | --- | --- | --- | --- | --- |
| Training cohort | Clinical model | 0.85 | 0.79-0.92 | 0.76 | 0.78 | 0.70 |
|  | Radiomics model | 0.79 | 0.71-0.87 | 0.74 | 0.82 | 0.61 |
|  | Clinical-radiomics model | 0.92 | 0.88-0.96 | 0.84 | 0.88 | 0.77 |
|  | PHE-volume-clinical model | 0.91 | 0.87-0.96 | 0.82 | 0.86 | 0.75 |
|  | PHE-Hematoma-clinical model | 0.91 | 0.86-0.95 | 0.82 | 0.86 | 0.75 |
| Test  cohort | Clinical model | 0.85 | 0.74-0.95 | 0.82 | 0.85 | 0.75 |
|  | Radiomics model | 0.71 | 0.57-0.86 | 0.61 | 0.83 | 0.46 |
|  | Clinical-radiomics model | 0.91 | 0.84-0.99 | 0.84 | 0.84 | 0.82 |
|  | PHE-volume-clinical model | 0.84 | 0.74-0.93 | 0.74 | 0.8 | 0.62 |
|  | PHE-Hematoma-clinical model | 0.90 | 0.82-0.98 | 0.75 | 0.78 | 0.69 |
