# Supplementary figures and images for: Radiomics features from perihematomal edema for prediction of prognosis in the patients with basal ganglia hemorrhage
Source: Front Neurol. 2022 Nov 8;13:982928. doi: 10.3389/fneur.2022.982928 (PMC9680901; doi:10.3389/fneur.2022.982928)

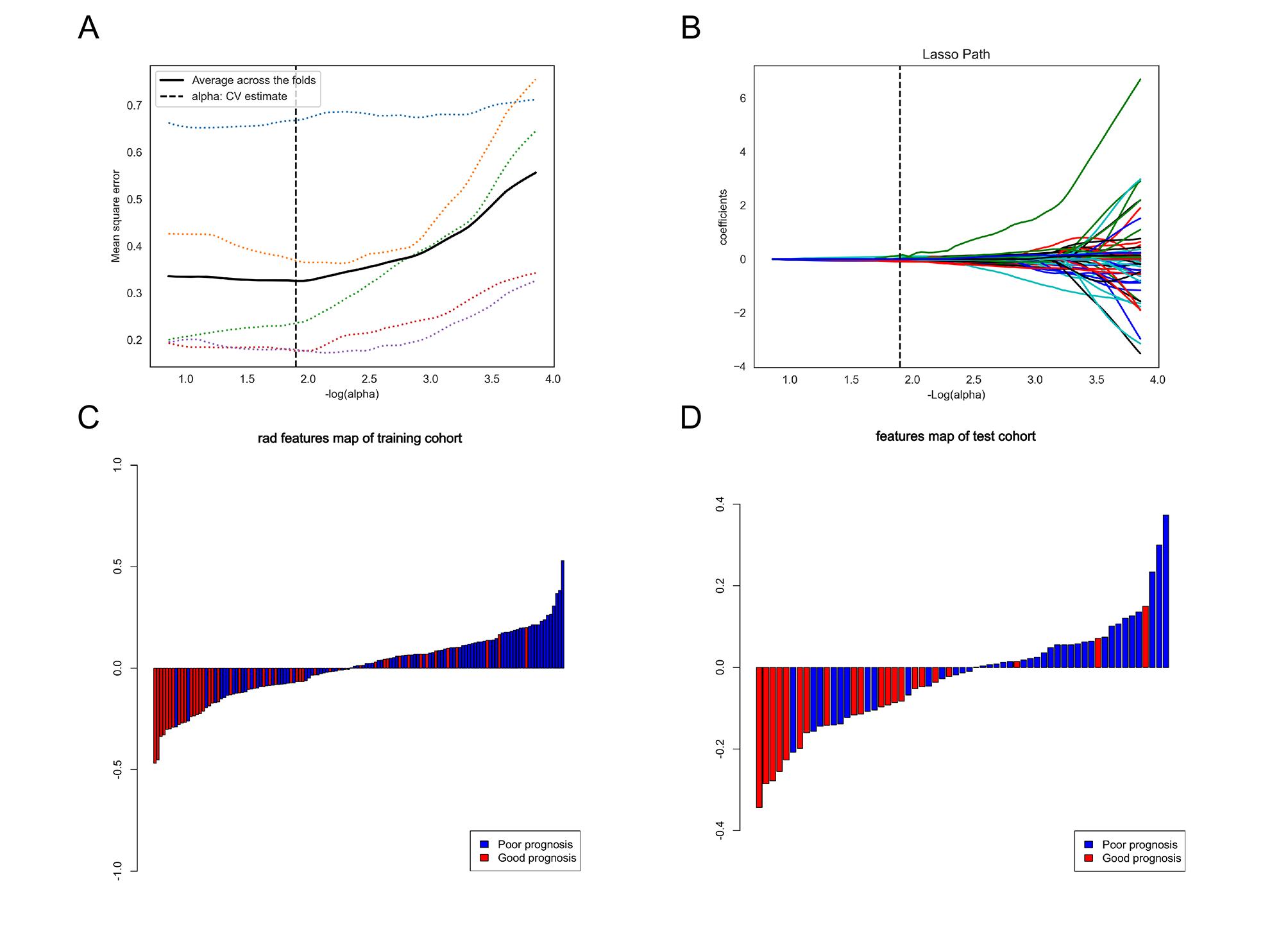

Supplement: Supplementary file 4 [file Image_1.TIFF]

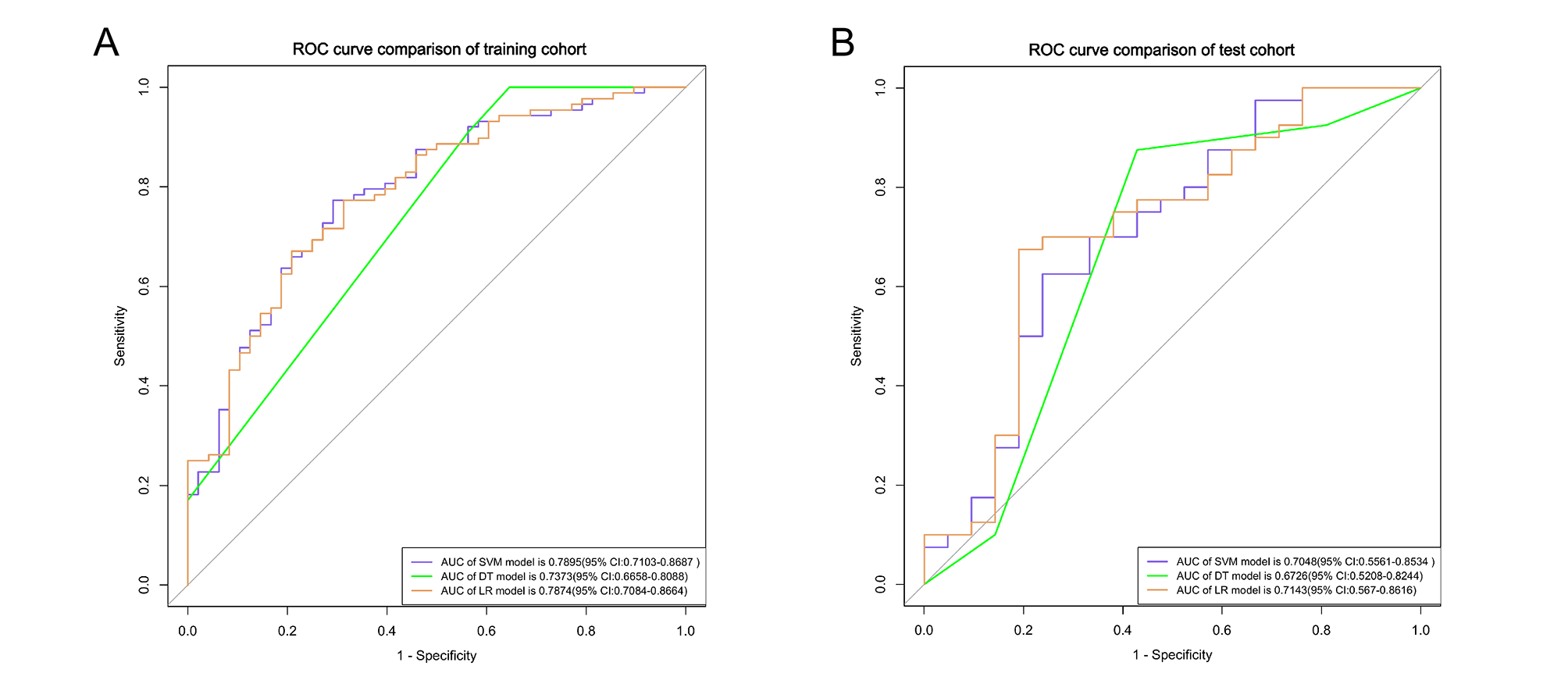

Supplement: Supplementary file 5 [file Image_2.TIFF]

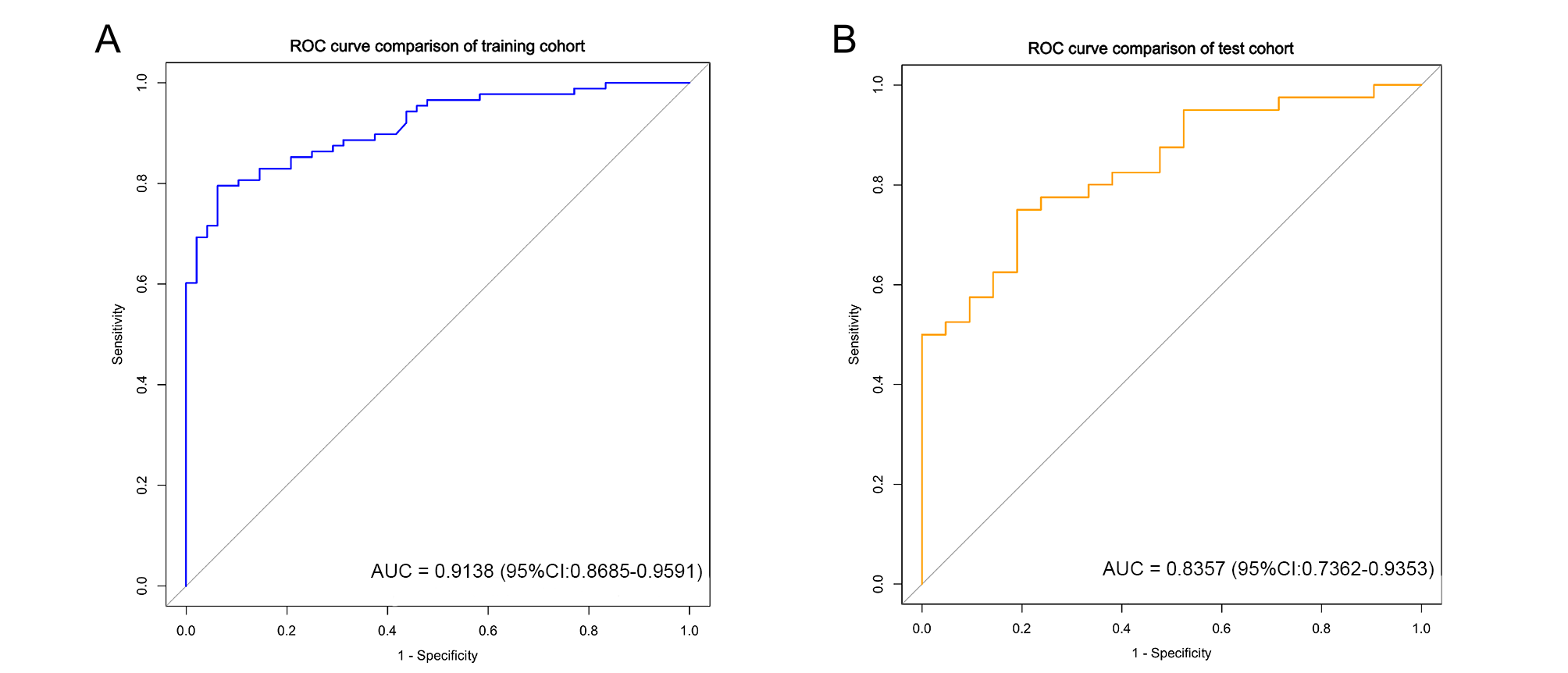

Supplement: Supplementary file 6 [file Image_3.TIFF]

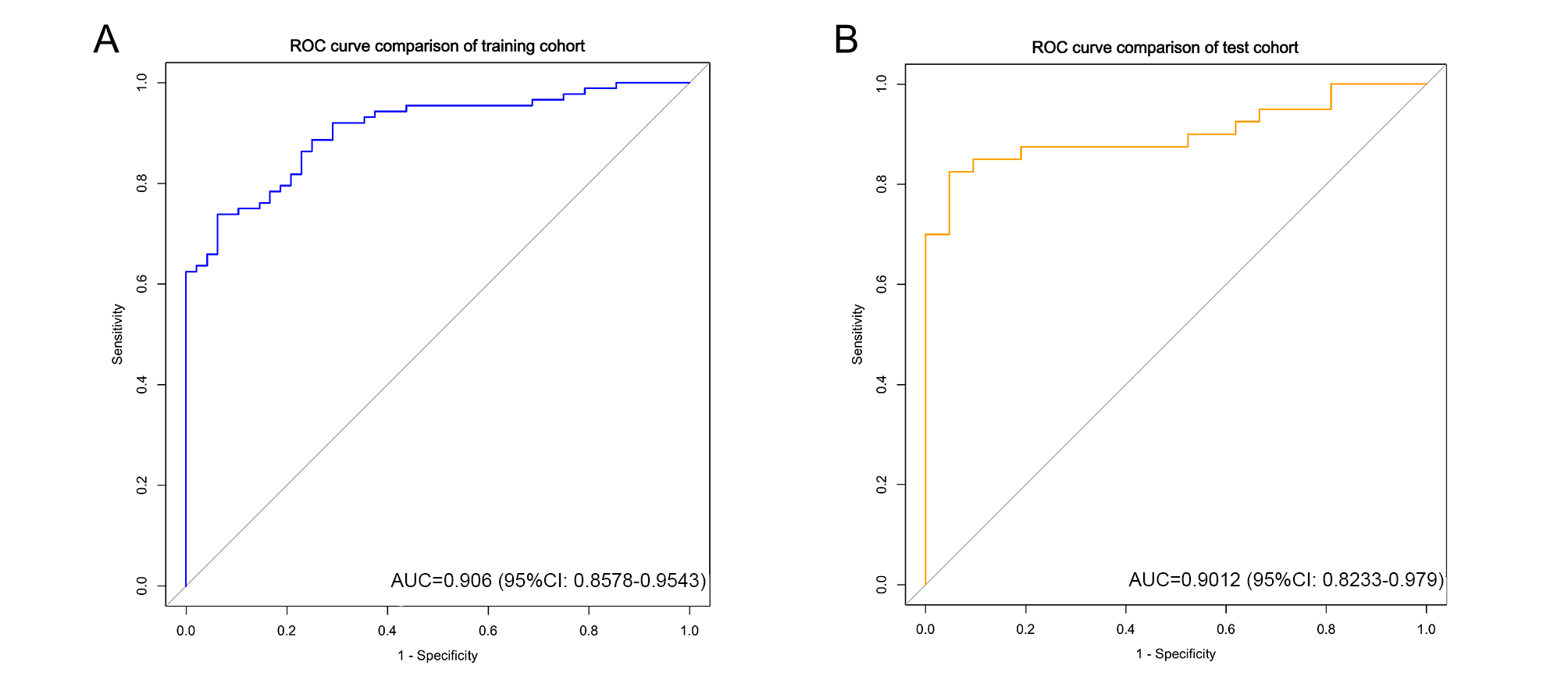

Supplement: Supplementary file 7 [file Image_4.TIF]
